# Results of the Treatment of Mental Conditions
*A paper read at the meeting of the Bristol Medico-Chirurgical Society on Wednesday, 13th November, 1935.


**Published:** 1935

**Authors:** G. de M. Rudolf

**Affiliations:** Medical Superintendent, Brentry Colony, Bristol


### RESULTS OF THE TREATMENT OF MENTAL CONDITIONS.*

BY

G. de M. Rudolf, M.R.C.P., D.P.M., D.P.H.,

*Medical Superintendent, Brentry Colony, Bristol.*

---

The inter-relations of the various bodily processes and the inter-relations of the bodily and mental processes are so complex, so intricate, and so involved, that successful results in the treatment of mental disorders can be obtained by entirely different methods. These methods may be purely physical, purely psychological, or a combination of the two. Whatever treatment is employed, the results obtained are frequently dependent upon the accuracy of the application of the treatment and the psychological skill and insight of the attending practitioner.

Many of the apparently different treatments produce the same ultimate chemical change. An example is seen in the many treatments advised for dementia præcox or schizophrenia. If untreated, this condition improves in about 20 per cent. of cases. Treatments that have been reported to increase this rate are salicylates, aseptic meningitis, sodium nucleinate, sulfosin, manganese chloride, occupational therapy, malaria, Ringer-Locke solution, somnifen, calcium, removal of sepsis, assisted respiration,

* A paper read at the meeting of the Bristol Medico-Chirurgical Society on Wednesday, 13th November, 1935.

vitamin D, hæmotherapy, psycho-analysis, ovary transplants and others.

Many of the successful treatments in the above list act upon the ratio of the intra-corpuscular sodium chloride to the intra-corpuscular sodium bicarbonate, some treatments tending to increase the bicarbonate, others to decrease the chloride. (Table 1.) The

TABLE 1. SCHIZOPHRENIA.

SUGGESTED HYPOTHESIS FOR MODE OF ACTION OF TREATMENTS.
(RATIO OF SODIUM BICARBONATE TO CHLORIDES IS INTRA-CORPUSCULAR.)[13]

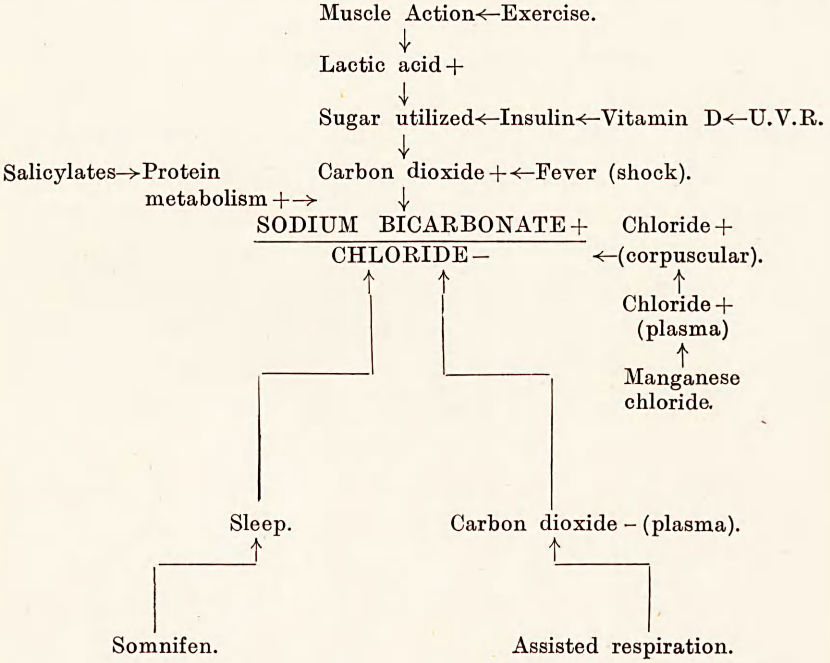

treatments are different methods for obtaining the same results.

Perhaps some of the most striking mental changes produced by physical methods are those occurring during the malarial treatment of general paralysis. Improvement of confusion, of amnesia, of disorientation is seen after recovery from delirium or the removal of cerebral tumours, but the gradual progressive diminution in the intensity of grandiose delusions is of greater interest. Table 2 shows the progressive

TABLE 2. MALARIAL TREATMENT OF GENERAL PARALYSIS.

WEEKLY SALARY GENERAL PARALYTIC STATED HE COULD EARN AS COMEDIAN ON MUSIC HALL STAGE. PREVIOUS OCCUPATION, KENNEL-MAN.[12]

| Date. | Salary. |
|---|---|
| 25th March, 1924 | £250 |
| 28th March, 1924 | £100 |
| 4th April, 1924 | £1,500 |
| 26th April, 1924 | £8,000 |
| 20th May, 1924 | Rigors ceased. |
| 1st June, 1924 | £500 |
| 5th June, 1924 | £300 |
| Previous to 3rd July, 1924 | £8 |
| 24th July, 1924 | See text. |

increase of intensity of delusions before the onset of the malaria, followed by a progressive decrease after the fever. The patient was questioned on the dates mentioned, but later, on 3rd July, he became irritable, whilst on 24th July he stated his idea of going on the stage "was all a delusion." When questioning the patient similar wording was used so as to avoid suggestion as much as possible. The delusions of another patient are seen in Table 3. Again there is a progressive decrease, although not expressed numerically.

Child guidance is performed, for good or ill, by every adult consciously or unconsciously. Guidance of the child is performed particularly by children's homes and societies. In addition, one Society, the

TABLE 3.

PROGRESSIVE DECREASE OF DELUSIONS FOLLOWING MALARIAL TREATMENT OF GENERAL PARALYSIS.[12]

| | | |
|---|---|---|
| 11th January, 1924 | .. | Promised position of Postmaster-General. |
| 3rd March, 1924 | .. | Rigors ceased. |
| 15th March, 1924 | .. | Could be made Postmaster-General if he appealed to the King. |
| 22nd March, 1924 | .. | Thinking of applying for Postmaster-General on leaving hospital. |
| 29th March, 1924 | .. | Decided not to apply. |
| 5th April, 1924 | .. | As above. |

Later stated previous ideas " ridiculous."

Waifs and Strays, has a home, in memory of the Society's founder, for the maladjusted child admitted to the ordinary homes.

Although this work is guidance of the child, the usual implication of the term " child guidance " is the guidance of psychologically abnormal children. Results of the work of clinics are gratifying. Burns,[5] giving the results for the Birmingham Clinic, states that of 160 cases treated in the first two years 110 (69 per cent.) were adjusted sufficiently to cease attendance, and 31 (17 per cent.) were partly adjusted. The type of case treated consisted of (*a*) personality traits (nervousness, fears, seclusiveness, etc.), (*b*) nervous habits (enuresis, stammering, habit spasms, etc.), (*c*) backwardness, (*d*) delinquency, and (*e*) behaviour problems.

At the clinic at Guy's Hospital Hardcastle[6] reports that of 60 children examined two years after cessation of attendance 57 per cent. were improved or much improved. Subsequently somewhat similar results were obtained in a group of 67 children, 64 per cent. being improved. In New York 73 per cent. of 197 children attending the Institute of Child Guidance were much improved (Witmer).[17] Therefore, good results are obtained in about two-thirds of the children attending child guidance clinics.

The improvement - rate in adults attending psychological clinics is also about the same. At the Institute of Medical Psychology about 60 per cent. of the patients are permanently relieved or cured (Rees).[11] Of 500 cases treated 65·6 per cent. were relieved (*i.e.* much improved or improved) at the time of discharge, and 55 per cent. three years later.

Table 4 shows the varying rates of improvement amongst different types of cases. In obsessional states the percentage of those relieved three years after discharge was inversely proportional to the length of treatment, the cases given less than 20 interviews showing a higher relieved-rate than those given over 60 interviews. For all cases treated the relieved rate three years after discharge was the same for those who received less than 20 interviews, for those who received from 20 to 60 interviews, and for those who received over 60 interviews. Neither sex nor civil state affected the relieved rate three years after discharge. The ages of the patients varied from 17 years to over 50 years, but in each of 5 age-groups the relieved rate three years after discharge varied from 50·6 to 60·5 per cent. The type of treatment used consisted of comparatively short methods, such as re-education with explanation of symptoms, although deep analysis, suggestion and hypnosis were all used. At Guy's Hospital Hardcastle[6] reports that of 33 cases treated 39 per cent. remained well two years after treatment.

Neustatter[10] treated 50 cases at the Maudsley Hospital. The methods used were analysis by Stekel's method (10 cases, 70 per cent. improved), psychological

TABLE 4.

RESULTS OF TREATMENT AT INSTITUTE OF MEDICAL PSYCHOLOGY. (LUFF AND GARROD.)[8]

| Diagnosis. | No. of Cases. | Condition on Discharge. | | | | Condition after Three Years. | | | | | | | Percentage Relieved. | |
|---|---|---|---|---|---|---|---|---|---|---|---|---|---|---|
| | | Relieved. | | | | Relieved. | | | | | | | | |
| | | Much Improved. | Improved. | Slightly Improved. | Not Improved. | Much Improved. | Improved. | Slightly Improved. | Not Improved. | Further Treatment at Institute. | Further Treatment Elsewhere. | Died. | On Discharge. | After Three Years. |
| Anxiety states .. | 220 | 72 | 104 | 30 | 14 | 74 | 67 | 11 | 32 | 8 | 22 | 6 | 80·0 | 64·0 |
| Hysteria .. .. | 75 | 13 | 25 | 17 | 20 | 17 | 21 | 6 | 16 | 1 | 14 | — | 50·6 | 50·6 |
| Obsessional states | 60 | 9 | 25 | 14 | 12 | 19 | 12 | 1 | 17 | 8 | 3 | — | 56·7 | 51·7 |
| Depressive states | 30 | 5 | 11 | 5 | 9 | 10 | 5 | 2 | 7 | — | 3 | 3 | 53·3 | 50·0 |
| Sexual difficulties | 30 | 6 | 16 | 4 | 4 | 9 | 13 | — | 3 | 2 | 3 | — | 73·3 | 73·3 |
| Paranoid states.. | 20 | 1 | 7 | 2 | 10 | 3 | 4 | — | 10 | — | 3 | — | 40·0 | 35·0 |
| Delinquency .. | 20 | 3 | 9 | 3 | 5 | 1 | 5 | 3 | 9 | — | 2 | — | 60·0 | 30·0 |
| Percentage .. | | 22·8 | 42·8 | 17·4 | 17·0 | 28·0 | 27·0 | 5·2 | 23·0 | 3·8 | 11·2 | 1·8 | 65·6 | 55·0 |

talks (23 cases, 52 per cent. improved), reassurance and medicine (15 cases, 73 per cent. improved), and suggestion (2 cases, both improved). Of 24 cases of anxiety 68 per cent. improved, and of 11 cases of depression 82 per cent. improved.

Skottowe and Lockwood,[15] reviewing 150 patients attending a psychiatric out-patient clinic, report that only 36 were treated as out-patients and followed up, 28 refused treatment or advice, and 17 were not followed up. Of the 36 treated and followed up 28 (77 per cent.) were recovered or much improved from two months to twenty months after first coming under observation.

A valuable study of psychoanalysis has been made by Kessel and Hyman.[7] Seven psychotics and 26 maladjusted cases were submitted to competent analysed analysts. The results were bad in 16 cases (48 per cent.), including the 7 psychotics. Of the 4 cases that became symptom free the authors considered that the improvement might be due to sexual satisfaction as much as to the analysis. Five cases showed incomplete cure and 8 were cured, 39 per cent. being improved or cured. Of the cures, in 3 the analyst produced the desired result without reaching the conclusion of the analysis.

Vocational guidance is proving of value. Of 100 boys and girls guided by the National Institute of Industrial Psychology,[9] the probability of success in those who followed the Institute's advice was 11 to 1 in favour, and correct predictions were made in nearly 80 per cent. of cases. Amongst 400 Borstal boys 69·5 per cent. of those placed in the work parties recommended by the Institute became Grade "A" workers, whereas only 45·6 per cent. of those recommended by their own house-masters became Grade "A" workers.

Psychotics show a comparatively high rate of improvement. For England and Wales for the year 1934 8,622 patients were discharged from mental hospitals as recovered, that is 32·1 per cent. of the admissions. In addition 5,734 patients were discharged relieved, that is 21·4 per cent. of the 26,819 admissions. Thus 53·5 per cent. of the admissions to mental hospitals improve sufficiently to leave the institutions.[2]

Wootton, Armstrong, and Lilley[18] followed up 131 cases of 156 discharged from Ewell Mental Hospital. Table 5 is drawn up from their results.

TABLE 5.

CASES DISCHARGED FROM EWELL MENTAL HOSPITAL (WOOTTON, ARMSTRONG AND LILLEY).[18]

| No. Discharged. | Remained Well. | Years after Discharge. |
|---|---|---|
| | Per cent. | |
| 16 | 44 | 2 |
| 26 | 73 | 3 |
| 45 | 62 | 4 |
| 44 | 66 | 5 |

Two-thirds of the cases discharged as recovered or relieved have undergone no relapse up to five years after leaving the Mental Hospital. Of schizophrenic patients 50 per cent. of 38 remained well five years after discharge, and 46 per cent. of those admitted were discharged. Of 24 puerperal cases 16 (67 per cent.) were discharged, 8 remaining well from two to five years later.

Varying rates of improvement have been recorded for dementia præcox or schizophrenia, but a rate of about 20 per cent. is the usual for untreated cases. For treated cases in series of 20 or more rates varying from 23 to 75 per cent. have been reported for different treatments. (Table 6.)

TABLE 6.

TREATMENTS OF SCHIZOPHRENIA.[13]

| Method. | No. of Cases. | Improvement per cent. | Authors. |
|---|---|---|---|
| Salicylates .. .. | 76 | 75 | Margulies (1927). |
| Aseptic meningitis | 64 | 57·8 | Carroll, Barr, Barry and Matzke (1925). |
| Sodium nucleinate | 32 | 39·7 | Donath (1913), Lundval. |
| Sulfosin.. .. .. | 59 | 39 | Schroeder (1929), Salinger (1929), Marcuse and Kallmann (1929). |
| Manganese chloride | 308 | 35·26 | English (1929), Reed (1929), Schrijer, Reiter and Bisgaard (1927). |
| Occupational therapy. | 20 | 35 | Main (1923). |
| Malaria .. .. | 48 | 33·3 | Yakubovsky (1929), Rudolf (1927), Wizel and Markuszewicz (1927). |
| Ringer-Locke Solution. | 49 | 28·6 | Morowoka (1927), Ikeda (1926), Sato and Morita (1918). |
| Somnifen .. .. | 26 | 25–33 | Kläsi (1922). |
| Calcium .. .. | 30 | 23·3 | Dodel, Rudolf, Bernolt and Kolle (1926). |
| Removal of sepsis | 35 | 22·8 | Kirby (1923), Holmes (1921). |

Cases of manic-depressive psychosis frequently improve, but many relapse again. Occasionally marked and sudden improvements occur after many years of disorder. In one such case a woman who had been in a condition of sub-acute mania for many years suddenly, for no apparent reason, became normal, except that she still wished to work in the acute ward as the convalescent wards were too quiet for her !

Prolonged narcosis with somnifen-insulin-glucose therapy has been stated to produce some improvement, Ström-Olsen and M. McCowan[16] reporting 38 per cent. of recoveries and 29 per cent. of improvements in 45 cases of manic-depressive psychosis. In a small series of 13 cases of psychoneuroses 62 per cent. of recoveries and 15 per cent. of improvements occurred.

The mental conditions associated with epilepsy usually show little change under treatment, although great improvement in the frequency of the fits may be produced. A progressive mental deterioration may set in. Its march cannot be stopped.

There is no known treatment for secondary dementia.

When an unselected series of general paralytics is treated with malaria from 25 to 33 per cent. undergo good remissions. Driver, Gammel and Karnosh, collecting 2,336 cases from the literature, found that 27·5 per cent. showed complete remissions and 26·5 per cent. incomplete remissions. The earlier the treatment the greater is the probability of a good remission. Four or five years after treatment the number of cases undergoing good remissions decreases until, at about the tenth year, the good remissions number about 11 to 20 per cent. of those treated. Even this is an improvement upon the 2 per cent. of spontaneous remissions occurring in non-treated cases.

In individual cases methods other than malaria produce excellent results, but the proportion of good remissions obtained is less than when malaria is used.

At present no change can be produced in the intelligence of the adult mental defective. Before the mind ceases to develop improvement occurs continually but at a lower level than normal. Once development has ceased no treatment can advance the intelligence, it can only develop that which is present. Improvement in the behaviour of the defective can, however, be brought about. As the defective is usually under care for abnormalities of behaviour, such improvement may permit the patient to leave a Colony and return to his home or go to guardians. On 1st January, 1935, 2,094, or 5·9 per cent., of 35,512 defectives in institutions in England and Wales were on licence away from the institutions. Of males 6·1 per cent. were on licence, and of females 5·5 per cent. These patients had improved sufficiently in their behaviour to be able to lead a life outside an institution.

At Brentry Colony[3] there is a system of grouping according to behaviour, so that the longer the period during which a patient has behaved well the greater his freedom. After three months' good behaviour the patient can obtain a pass allowing him to go to his work, be at his work, and return from his work without observation by the staff. After six months' good behaviour he can be admitted to a privilege block and is allowed parole outside the estate with two others; after nine months' good behaviour he can be admitted to another block with additional privileges and has individual parole upon the estate, and after twelve months' good behaviour the patient can be admitted to blocks managed by committees of defectives elected by the patients in these blocks. This graded series of freedom has been in existence since 1932, and appears to have increased the numbers of patients on licence, as the licence rate has increased from 10·8 per cent. of the population for the years 1929–31 to 16·2 per cent. for the year 1934. There has been no alteration in the arrangements for the finding of homes to which the patients go. For 1934 those proceeding on licence were 88·1 per cent. of the number of admissions. A summary of the results of treatment is shown in Table 7.

This brief summary of the results of treatment of

TABLE 7.

RESULTS OF OUT-PATIENT AND IN-PATIENT TREATMENT.

| | No. of Cases. | Improved per cent. | Period after Treatment. | Author or Institution. |
|---|---|---|---|---|
| Child guidance. | 160 | 86 | At termination. | Burns. |
| | 197 | 73 | At termination. | Witmer. |
| | 60 | 57 | 2 years. | Hardcastle. |
| | 67 | 64 | 2 years. | Hardcastle. |
| Vocational guidance. | 400 | 69·5 | — | National Institute of Industrial Psychology. |
| | 100 | nearly 80 | — | |
| Psychological Clinics. (Adult.) | 500 | 65·6 | At termination. | Luff and Garrod. |
| | 50 | 62 | At termination. | Neustatter. |
| | 500 | 55 | 3 years. | Luff and Garrod. |
| | 33 | 39 | 2 years. | Hardcastle. |
| | 36 | 77 | 2 to 20 months. | Skottowe and Lockwood. |
| Mental Hospitals. | Admissions. 26,819 | 53·5 | — | England and Wales. |
| | 317 | 49·2 | — | Wootton, Armstrong and Lilley. |
| Mental Deficiency Institutions. | 48 | 88·1 | — | Brentry. |
| | Population. 355 | 16·2 | — | Brentry. |
| | 35,512 | 5·9 | — | England and Wales. |

mental conditions is incomplete without some thoughts upon the future. What are the possible methods of future treatment ?

The regular ten per second electrical waves of the Berger rhythm abolished or diminished by a slight degree of visual stimulation or by a marked degree of other forms of attention (Adrian and Matthews[1]) ; the cerebral currents altered by the application of sensory stimuli to the limbs with production of the currents of action (Sarkisoff),[14] and the 20 per cent. cessation of epileptic fits obtained with cerebral diathermy (Brünner-Ornstein)[4] all point to the possibility of the successful treatment of abnormal mental conditions by active electrical technique.

### REFERENCES.

1 Adrian, E. D., and Matthews, B. H. C., *Brain*, 1934, lvii. 355 (*Brit. Med. Jour.*, 1935, i. 592.)

2 Board of Control, *Annual Report for* 1935, Part I, 16.

3 Brentry Colony, *Annual Report for* 1934–35, pp. 20, 25.

4 Brünner-Ornstein, M., *Abstracts*, Second International Neurological Congress, 1935, p. 134.

5 Burns, C. L. C., *Mental Welfare*, 1935, xvi. 1.

6 Hardcastle, D. H., *Jour. Ment. Sc.*, 1935, lxxx. 536.

7 Kessel, L., and Hyman, H. T., *Jour. Amer. Med. Assoc.*, 1933, ci. 1,612. (*Brit. Med. Jour.*, 1933, ii. 1,175.)

8 Luff, M. C., and Garrod, M., *Brit. Med. Jour.*, 1935, ii. 54.

9 National Institute of Industrial Psychology, *Brit. Med. Jour.*, 1935, i. 256.

10 Neustatter, W. L., *Lancet*, 1935, i. 796.

11 Rees, J. R., *Brit. Med. Jour.*, 1935, i. 855.

12 Rudolf, G. de M., *Therapeutic Malaria.* London, 1927.

13 Rudolf, G. de M., *Jour. Ment. Sc.*, 1931, lxxvii. 767.

14 Sarkisoff, S., *Abstracts*, Second Neurological Congress, 1935, p. 100.

15 Skottowe, I., and Lockwood, M. R., *Jour. Ment. Sc.*, 1935, lxxxi. 502.

[16] Ström-Olsen, R., and McCowan, M. L. M., *Jour. Ment. Sc.*, 1934, lxxx. 658.

[17] Witmer, H. L., 1933, " The Outcome of Treatment in a Child Guidance Clinic," Smith College Studies of Social Work (cited by Hardcastle).

[18] Wootton, L. H., Armstrong, R. W., and Lilley, D., *Jour. Ment. Sc.*, 1935, lxxxi. 168.

